# Relationship between the expression of hTERT and EYA4 mRNA in peripheral blood mononuclear cells with the progressive stages of carcinogenesis of the esophagus

**DOI:** 10.1186/1756-9966-28-145

**Published:** 2009-11-25

**Authors:** Hao Li, Tao Yu Diao, Zhi Ying Zhou, Fang Yan Yang, Qing Ma, Qing Hui Li

**Affiliations:** 1 Tumor Center, Qilu Hospital, Shandong University, Jinan, 250012, PR China; 2 Department of Epidemiology, School of Public Health, Shandong University, Jinan, 250012, PR China; 3 School of economics of Shandong University, Jinan, 250012, PR China; 4 Department of Epidemiology, Institute of Basic Medicine of Shandong Academy of Medical Sciences, Jinan, 250062, PR China

## Abstract

**Objective:**

To establish a relationship between esophageal squamous cell diseases and the expression of human telomerase reverse transcriptase (hTERT) and Eyes absent 4 (EYA4) mRNA in peripheral blood mononuclear cells.

**Methods:**

Subjects were 50 patients with esophageal squamous cell carcinoma (ESCC), 50 with dysplasia (ESCD), 50 with basal cell hyperplasia (BCH) and 50 controls. All subjects were residents of Feicheng County, Shandong Province, China , diagnosed by histopathology. Expression of hTERT and EYA4 mRNA in peripheral blood was determined by semiquantitative reverse transcription-polymerase chain reaction (RT-PCR).

**Results:**

The hTERT and EYA4 mRNA positive expression increased according to disease severity. At the cut-off value of ≥ 0.2, the positive expression rates of EYA4 were 14% for controls, 20.0% for BCH, 26% for ESCD and 52% for ESCC, respectively. At the cut-off value of ≥ 0.8, the positive expression rates of hTERT in the four groups were 24%, 30.0%, 52% and 80%, respectively. Using a positive value of 0.47 for EYA4, the testing sensitivities in the ESCD and ESCC groups were 4% and 16%, respectively, and the testing specificity increased to 100%. Using a positive value of 1.0 for hTERT, the testing sensitivities in the ESCD and ESCC groups were 48% and 60%, respectively, and the testing specificity increased to 72%. The testing sensitivities in the predicting ESCD and ESCC in the discriminant model including EYA4 and hTERT and the five traditional risk factors (sex, age, smoking, alcohol drinking, and family history of esophageal cancer) were 70% and 80%, and testing specificities were 76% and 88% respectively. However, the testing sensitivities and specificities in the predicting ESCD and ESCC in the model only including the above five traditional risk factors were lower than that in the former case.

**Conclusion:**

EYA4 and hTERT mRNA expression increased with the severity of esophageal pathological changes and may be useful for identifying high-risk endoscopy candidates or for monitoring changes in premalignant esophageal lesions.

## Background

Cancer cells release protein markers into the peripheral blood, but these are difficult to detect in the serum at the early stage of cancer. However, in the peripheral blood, circulating mononuclear cells may phagocytose cancer or precancer cells and, thereby, express epithelial markers within their phagocytosed contents. It is possible that tumor markers will show up in mononuclear cells before they themselves could be detected in the circulation. Therefore, mRNA expression of the genotype of these cells, in theory, can improve the sensitivity of detection of early cancers.

The human telomerase reverse transcriptase (hTERT gene) mRNA expression is the most general molecular marker for the identification of human cancer and can be detected in 85% of all tumors, whereas most healthy tissues exhibit little or no expression [1-4]. In healthy esophagus tissue, hTERT expression is predominantly localized in the basal cell layers of the columnar epithelium [5]. Differential hTERT expression between tumor tissues and healthy tissues makes this gene a promising marker for the detection of tumor cells [6,7].

Eyes absent 4 (EYA4) is one of four members of the EYA gene family that is homologous to the eyes absent gene in Drosophila [8-10]. Eyes absent works as a key regulator of ocular differentiation and may also modulate apoptosis. Recently, the value of methylated EYA4 as a marker for Barrette's esophagus and esophageal adenocarcinoma has been suggested [11,12]. However, to our knowledge, no reports have yet linked expression of the EYA4 gene linkage with esophageal squamous cell carcinoma (ESCC).

Endoscopic screening with Lugol dye and pathologic evaluation are useful screening tools for early stage esophageal cancer and for ascertaining the different stages of esophageal carcinogenesis in populated areas with high incidence [13]. However, the lack of strict scientific methods for determining high-risk persons who should undergo endoscopic testing, and the resulting cost-efficiency issues, currently impede this type of screening. Even in areas with high incidence of ESCC, the detection rate of ESCC *in situ *or at early stage is very low. A crucial requirement is a reliable method for distinguishing healthy persons and high-risk persons in need of an endoscopic test.

For this reason, in the present study, our aim was to establish a relationship between the expression of hTERT and EYA4 mRNA in peripheral blood mononuclear cells, which may phagocytose cancer or precancer cells and express epithelial markers within their phagocytosed contents, with the progressive stages of carcinogenesis of the esophagus. The overall goal is to develop an evaluation criterion that will allow persons living in high-incidence cancer areas and at high risk for ESCC to be included in endoscopic screening programs.

## Methods

### Subjects

The subjects consisted of 50 patients diagnosed with ESCC (12 *in situ *and 38 invasive carcinomas), 50 cases with esophageal squamous cell dysplasia (ESCD), 50 cases with basal cell hyperplasia (BCH), and 50 controls in the endoscopic screening program from January 2004 to December 2006 in Feicheng county, China. Any patients with history of nephrosis, dermatosis, lung and head-and-neck diseases, liver diseases, diabetes, or cardiovascular diseases including coronary heart disease, angina pectoris, myocardial infarction, cardiac arrhythmia, heart failure diagnosed via general medical check, electrocardiogram and abdomen supersonic inspection were excluded.

All subjects took part in the screening program by undergoing an endoscopic staining examination with 1.2% iodine solution, and biopsies of the subjects were taken from non-staining areas of mucosa. Two pathologists took the biopsies of mucosa for separate pathologic evaluation. Fifty controls that had non-staining areas of mucosa and diagnosed as normal mucosa were also included.

The study protocol was approved by the Shandong Academy of Medical Sciences Ethics Committee and an informed consent was obtained from each subject. A questionnaire form was used to interview all of the subjects and included sociodemographic characteristics, alcohol use, tobacco use, and family history of esophageal cancer. A 4 ml peripheral vein blood sample was drawn into sterile cryovials containing 0.5 ml anticoagulation reagent. The blood samples were stored at -70°C until used for assays. In the ESCC group, 20 specimens of ESCC tissues were obtained for testing the correlation of hTERT and EYA4 mRNA expression in peripheral blood mononuclear cells with that in ESCC tissues.

### RT-PCR of hTERT and EYA4 from peripheral blood

Total RNA was extracted from peripheral blood mononuclear cells by the acid guanidium-isothiocyanate-phenol-chloroform method.

The primers for hTERT were 5'-ACC GTC TGC GTG AGG AGA TC-3' and 5'-CCG GTA GAA AAA GAG CCT GTT C-3'.

The primers for EYA4 were 5'-TCC CCA CAG CTG TAT CCT TC-3'and 5'-AAC TGA GGC AGC CAC TCT GT-3' [12]. The quality of RNA and cDNA synthesis was ascertained by amplification of human β-actin as an internal control.

The primers for β-actin were 5'-GTGGGGCGCCCCAGGCACCA-3' and 5'-CTCCTTAATGTCACGCACGATTTC-3' [14].

The primers amplified 131 bp, 250 bp, and 540 bp products from hTERT, EYA4, and β-actin, respectively.

RNA was reverse transcribed into cDNA using a First Strand cDNA Synthesis Kit (Promega, Madison, USA). After reverse transcription, 3 μl of synthesized cDNA was amplified in a 50 μl PCR reaction mix containing 20 mM (NH_4_)_2_SO_4_, 75 mM Tris-HCl (pH8.8), 0.01%Tween20, 2 mM MgCl_2_, 0.2 mM dNTP, 0.2 μM of each of the primers and 1 unit Taq DNA polymerase (Fermentas, Vilnius, Lithuania). The reaction was performed at 95°C for 5 min, followed by 35 cycles at 94°C for 1 min, 58°C for 1 min and 72°C for 1 min, and a final extension at 72°C for 7 min.

A negative control without template cDNA was performed with every PCR reaction. After PCR reactions, 10 μl of the PCR products were electrophoresed on a 1.2 percent agarose gel and visualized by ethidium bromide staining. The specificity of the PCR products was confirmed by direct sequencing. Band intensity of ethidium bromide fluorescence was measured using NIH Image Analysis Software Ver 1.61 (National Institute of Health, Bethesda, MD, USA). Bands intensities were determined by comparison to those of β-actin.

### hTERT and EYA4 RT-PCR in ESCC tissues

RT-PCR was also used to evaluate hTERT and EYA4 mRNA expression in 20 specimens of ESCC tissues sampled from the cancer group for confirmation of the accuracy of hTERT and EYA4 mRNA expression in peripheral blood. The RNA in the tissue was extracted by the same method as that described for the peripheral blood cells.

### Statistical Analysis

Pearson's χ^2 ^test was used to examine differences in sociodemographic characteristics, alcohol use, tobacco use, and family history of esophageal cancer among the cancer and control groups. Smoking index equals the number of cigarettes per day multiplied by smoking years. Alcohol drinking index equals the amount of alcohol drinking per month multiplied by drinking years. The association between the expression of hTERT and EYA4 mRNA and esophageal cancers was evaluated by odds ratios (ORs) and 95% confidence intervals (95% CIs), which were calculated using a multinomial logistic regression model after adjusting for the variables of age, smoking index and drinking index.

The sensitivity and specificity was calculated using the receiver operating characteristic (ROC) curves and the area under curve (AUC) for hTERT and EYA4 mRNA expression. The ratios of the band intensity of hTERT or EYA4 to β-actin are used the cut off values. The cut-off points of that were used in the discriminating between positive and negative status with the two biomarkers.

In order to determine high-risk people who need to take the endoscopic examination in the screening survey of esophageal lesions, the determinant regression model was used. In these models, hTERT and EYA4 combined with the risk factors including sex, age, smoking, alcohol drinking and family history of esophageal cancer, which were found by a traditional epidemiological case-control study in this area, are independent variables. The results of these model output will display the ability to distinguish cases and the normal controls. All statistical analyses were performed using SPSS version 15.0 software package (SPSS, Chicago, III).

## Results

### hTERT and EYA4 mRNA expression

Sociodemographic characters and possible risk variables in the cancer and control groups are summarized in Table 1. The univariate analysis showed significant differences in the percentages of age and alcohol drinking index between the cancer and control groups.

**Table 1:** Demographic characteristics and possible risk variables of the study subjects*

Variables	Control s (n = 50)	BCH (n = 50)	ESCD (n = 50)	ESCC (n = 50)
Gender, n(%)				
male	35(70.0)	35(70.0)	30(60.0)	30(60.0)
female	15(30.0)	15(30.0)	20(40.0)	20(40.0)
age(years), n(%)				
40~50	19(38.0)	19(38.0)	8(16.0)	7(14.0)
51~60	18(36.0)	18(36.0)	25(50.0)	23(46.0)
61~70	13(26.0)	13(26.0)	17(34.0)	20(40.0)
Smoking index, n(%)				
Never	24(48.0)	24(48.0)	25(50.0)	24(48.0)
1~600	13(26.0)	13(26.0)	14(28.0)	14(28.0)
≥ 600	13(26.0)	13(26.0)	11(22.0)	12(24.0)
Drinking index, n(%)				
Never	19(38.0)	19(38.0)	26(52.0)	25(50.0)
< 100	15(30.0)	15(30.0)	14(28.0)	8(16.0)
≥ 100	16(32.0)	16(32.0)	10(20.0)	17(34.0)
Family history of esophageal cancer, n(%)				
No	39(78.0)	39(78.0)	43(86.0)	44(88.0)
yes	11(22.0)	11(22.0)	7(14.0)	6(12.0)
Education:				
Illiterate or primary school	9(18.0)	8(16.0)	25(50.0)	37(74.0)
Junior high school and over	41(82.0)	42(84.0)	25(50.0)	13(26.0)
per capita annual income($)				
< 300	6(12.0)	2(4.0)	12(24.0)	19(38.0)
300-	16(32.0)	15(30.0)	8(16.0)	22(44.0)
≥ 600	28(56.0)	33(66.0)	30(60.0)	9(18.0)

*:There are significant differences of age, alcohol drinking index, education and per capita annual income among the four groups, and the values of Chi-square test are 29.044(*P *< 0.001), 13.974(*P *= 0.03), 48.436(*P *< 0.001) and 38.973(*P *< 0.001), respectively. Smoking index = cigarette/day × number of smoking years. Alcohol drinking index = ((white spirits(g) × 0.38+ wine (g) × 0.12+ beer(g) × 0.04)/month ×12)/365 day. BCH, Basal cell hyperplasia; ESCD, esophageal squamous cells dyspalsia; ESCC, esophageal squamous cells cancer.

The Spearman's correlation coefficient between hTERT and EYA4 was 0.385 (*P *< 0.05). The correlation coefficients between hTERT or EYA4 and the four groups were 0.484 and 0.213, respectively (*P *< 0.05).

**Figure 1:**
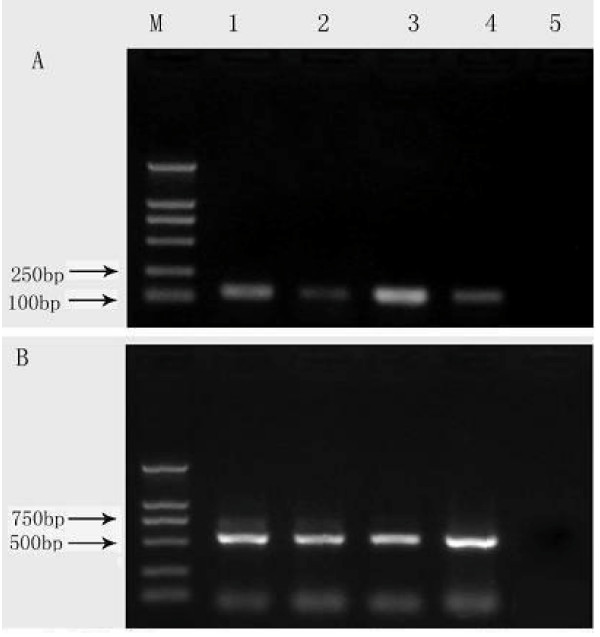
**Expression of hTERT mRNA in peripheral blood mononuclear cells among cases of esophageal squamous cell lesions and controls**. M: DNA ladders; lane 1: cases with basal cells hyperplasia; lane 2: normal controls; lane 3: cases with esophageal squamous cell carcinoma; lane 4: cases with esophageal squamous cell dysplasia; lane 5: negative control (no cDNA). The PCR products are 131 bp for hTERT(A) and 540 bp for β-actin (B).

**Figure 2:**
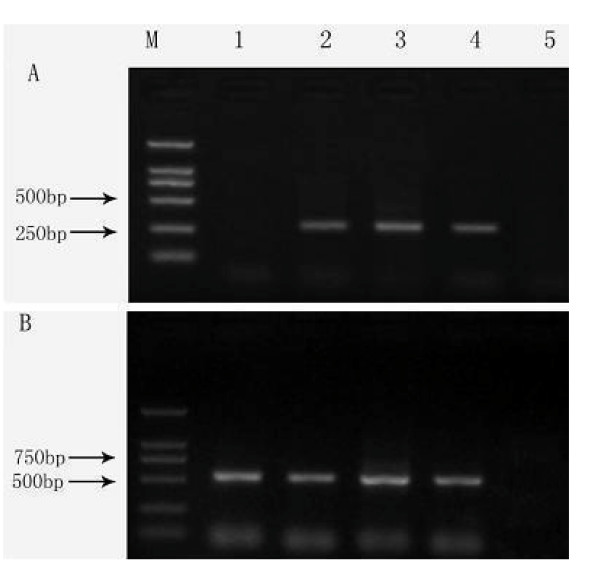
**Expression of EYA4 mRNA in peripheral blood mononuclear cells among cases of esophageal squamous cell lesions and controls**. M: DNA ladders; lane 1: normal controls; lane 2: cases with basal cells hyperplasia; lane 3: cases with esophageal squamous cell carcinoma; lane 4: cases with esophageal squamous cell dysplasia; lane 5: negative control (no cDNA). The PCR products are 250 bp for EYA4 (A) and 540 bp for β-actin (B).

The hTERT and EYA4 mRNA expression in the assay is shown in Table 2, Figure 1 and Figure 2. There was significant increase for the positive rates of hTERT or EYA4 mRNA expression in peripheral blood mononuclear cells with the progressive stages from normal cells to cancer in the esophageal carcinogenesis.

**Table 2:** Rates of detection of EYA4 and hTERT mRNA in peripheral blood mononuclear cells of the study subjects

	Control subjects (n = 50)	BCH (n = 50)	ESCD (n = 50)	ESCC (n = 50)
EYA4				
≥ 0.2, n(%)	7(14.0)	10(20.0)	13(26.0)	26(52.0)
< 0.2, n(%)	43(86.0)	40(80.0)	37(74.0)	24(48.0)
hTERT				
≥ 0.8, n(%)	12(24.0)	15(30.0)	26(52.0)	40(80.0)
< 0.8, n(%)	38(76.0)	35(70.0)	24(48.0)	10(20.0)

^×^:χ^2 ^= 19.643, *P *< 0.001, and linear-by-linear association = 16.246, *P *< 0.001 for EYA4 mRNA expression in the four groups; χ^2 ^= 69.149, *P *< 0.001 and linear-by-linear association = 41.994, *P *< 0.001 for hTERT mRNA expression in the four groups. BCH, Basal cell hyperplasia; ESCD, esophageal squamous cells dyspalsia; ESCC, esophageal squamous cells cancer.

As shown in Table 2, the band intensity ratios of EYA4 mRNA with a β-actin positive cut-off value of ≧ 0.2 indicated that EYA4 mRNA expression increased progressively according to the severity of the pathology: controls 14.0% (7/50), BCH 20.0% (10/50), ESCD 26.0% (13/50), and ESCC 52.0% (26/50). There was a significant linear-by-linear association of the four groups.

The band intensity ratios of hTERT mRNA with a β-actin positive cut-off value of ≧ 0.8 indicated that hTERT mRNA expression also increased with the progressively severity of the disease, and the positive expression rates in the four groups were 24% (12/50), 30.0% (15/50), 52% (26/50) and 80% (40/50), respectively.

The Spearman correlation coefficient of hTERT and EYA4 mRNA expression in peripheral blood mononuclear cells and in the tissues was 0.80 (P < 0.01). This indicated that the expression of the two markers in peripheral blood mononuclear cells was accurate.

As shown in Table 3, multinomial logistic regression analysis gave odds ratios (ORs) for EYA4 and hTERT mRNA expression also increased with the severity of the diseases after adjustment for age, gender, smoking index, drinking index and family history of esophageal cancer. However, only the OR value of the EYA4 mRNA expression in ESCC group was significant.

**Table 3:** Association of the expression of EYA4 and hTERT mRNA in peripheral blood mononuclear cells with esophageal diseases

	BCH (n = 50)	ESCD (n = 50)	ESCC (n = 50)
EYA4 mRNA			
OR(95%CI)	1.32(0.47-3.66)	1.85(0.69-4.94)	5.69(2.23-14.53)
OR(95%CI)^+^	1.90(0.62-5.81)	1.72(0.54-5.45)	5.07(1.56-16.52)
hTERT mRNA			
OR(95%CI)	0.90(0.33-2.45)	1.18(0.42-3.36)	2.03(0.63-6.55)
OR(95%CI)^+^	1.10(0.37-3.26)	1.12(0.34-3.72)	2.87(0.63-13.07)

+: OR(95%CI) was adjusted for age, smoking, drinking, income and family history of esophageal carcinoma. BCH, Basal cell hyperplasia; ESCD, esophageal squamous cells dyspalsia; ESCC, esophageal squamous cells cancer.

### The feasibility of prediction of high-risk persons

As shown in Table 4, using ratios of EYA4 mRNA expression to β-actin with a positive cut-off value of 0.47, testing sensitivities in ESCD and ESCC became 4% and 16%, respectively, and the testing specificity increased to 100%, where no false positive samples were existed in the study.

**Table 4:** The sensitivity and specificity of EYA4 and hTERT mRNA expression

		ESCC		ESCD		BCH	
		
item	Cut off level	Sensitivity (%)	Specificity (%)	Sensitivity (%)	Specificity (%)	Sensitivity (%)	Specificity (%)
hTERT							
	≥ 0.3	96.0	5.0	98.0	5.0	98.0	5.0
	0.5-	88.0	19.0	93.0.0	22.0	90.0	22.0
	1.0-	60.0	72.0	48.0	72.0	31.0	72.0
	1.5-	12.0	94.4	12.0	90.0	5.0	90.0
	AUC	0.820		0.671		0.566	
EYA4							
	≥ 0.20	76.0	64.0	36.0	64.0	12.0	64
	0.30-	40.0	73.0	27.0	73.0	0.0	73
	0.40-	20.0	90.0	10.0	90.0	0.0	90
	0.47-	16.0	100.0	4.0	100.0	0.0	100.0
	AUC	0.693		0.553		0.520	

NOTE. AUC:area under curve. The cut-off levels (the band intensity ratios of hTER or EYA4 to β-actin) written in bold are the cut-off points that used in the discriminating between positive and negative status with different markers. BCH, Basal cell hyperplasia; ESCD, esophageal squamous cells dyspalsia; ESCC, esophageal squamous cells cancer.

Using ratios of hTERT mRNA expression to β-actin with a positive cut-off value of ≥ 1.5, the testing sensitivities and specificities in ESCD and ESCC were 12% and 90%, 12% and 94%, respectively.

Table 5 showed the feasibility of prediction of high-risk persons. It is clear displayed when the hTERT and EYA4 mRNA expression and the traditional risk factors (sex, age, smoking, drinking, and family history of ESCC) included in the discriminat model 1 and model 3, the sensitivity and specificity was 80% and 88% for predicted ESCC, and 70% and 76% for predicted ESCD, respectively. These results were higher than the results of predicted ESCC and ESCD in the discriminat model 2 and model 4, including the above five traditional risk factors only. The results indicated that hTERT and EYA4 mRNA expression combined with the traditional risk factors are useful to set up a discriminating function model, which maybe used to determine a high-risk person needing to take the endoscopic testing in the high-incidence area. However, in these models, nearly half or more than half of all cases in each group were ungrouped in the analysis.

**Table 5:** The sensitivity and specificity for the positive expression of hTERT and EYA4 mRNA combing the traditional risk factors by discrimination analysis

Model	Original group	Predicted group membership		sensitivity	Specificity
**1**	Discrimination of ESCC/control:	control	ESCC		
	control	44	6	80.0%	88.0%
	ESSC	10	40		
	Ungrouped cases	54	46		

**2**	Discrimination of ESCD/control:	control	ESCC		
	control	38	12	64.0%	76.0%
	ESCC	18	32		
	Ungrouped cases	44	56		

**3**	Discrimination of ESCD/control:	control	ESCD		
	control	38	12	70.0%	76.0%
	ESCD	15	35		
	Ungrouped cases	27	73		

**4**	Discrimination of ESCD/control:	control	ESCD		
	control	39	11	64.0%	76.0%
	ESCD	18	32		
	Ungrouped cases	41	59		

Note: The discrimination model 1 and model 3 included variables of the hTERT and EYA4 and the five traditional risk factors (sex, age, smoking, alcohol drinking and family history of esophageal cancer). The discrimination model 2 and model 4 only included the five traditional risk factors. ESCD, esophageal squamous cells dyspalsia; ESCC, esophageal squamous cells cancer.

## Discussion

In a retrospective death survey carried out in the 1970s, Feicheng County was second only to Lin County of Henan Province as the area with the highest incidence of ESCC [15]. For the past 35 years, the mortality rate of ESCC has remained high in Feicheng County [16]. Epidemiological research has shown that there is a difference in the risk factors related to ESSC in the two areas [17,18].

We carried out a program of endoscope screening for esophageal lesions using 1.2% iodine staining between January 2004 and December 2006 in Fetching County. The study included all of the residents aged from 40 to 69, who agreed to participate in the program after explanation of the purpose of the study. Prior to this study, we had conducted a case-control study of esophageal cancer based on hospital data from Feicheng. This study found that esophageal cancer was associated with the risk factors of smoking, alcohol drinking and family history of the disease. In the screening explanation, we therefore especially encouraged those persons who were heavy smokers or drinkers, or who had a positive family history of esophageal cancer, to participate in the study and undergo endoscopic inspection [19].

Based on the screening data, we carried out another case-control study. There were 235 ESCC cases (70 early cancers identified in screening program, 183 were advanced cancer diagnosed in hospitalized patients) and 8159 controls who were confirmed clear by endoscopy and mucosal staining in the screening program. After adjusting for the three confounders (age, sex and education), we found that smoking and alcohol drinking were the top ranked risk factors for esophageal cancer. When smoking and alcohol drinking were combined, the *OR *was 2.73 (95%*CI*: 1.54-4.82), and the proportional attribute relative risk was 51.47 per cent for males. When smoking, alcohol drinking and family history of esophageal cancer were combined, the *OR *was 3.40 (95%*CI*: 1.68-6.89), and the proportional attribute relative risk was 15.4 per cent for males [20]. The risk factors identified in the study were consistent with the results of our previous case-control study based on hospital data.

Although there was no test fee charged for our screening survey, the response rate of residents participating was very low. The main reason was lack of a method to identify high-risk persons who may be suffering from esophageal premalignant diseases, and to persuade these persons to participate in the endoscopic examination.

Telomeres are nucleoprotein complexes that constitute the ends of eukaryotic chromosomes [21]. To maintain telomere length of telomerase is necessarily to indefinite proliferation of human cells. The human telomerase complex consists of human telomerase-associated RNA (hTR), providing the template for telomeric repeat synthesis, and human telomerase reverse transcriptase (hTERT), representing the catalytic subunit of the complex [22]. One Chinese study reported that hTERT mRNA positive expression was 96.6% (28/29) of ESCC, 48.9% (23/47) of dysplasia, and 7.5% (2/29) of normal tissues [23].

In our study, the positive rates of hTERT mRNA expression in peripheral blood mononuclear cells increased with the progressive stages of the esophageal carcinogenesis. However, it is clear that the positive expression rate of hTERT in peripheral blood mononuclear cells of the normal controls in our study is higher than that in the normal tissues of the above paper reported. Accordingly, Lord reported on higher hTERT levels in histological normal squamous esophagus tissues from cancer patients compared with hTERT levels found in normal esophageal tissues from patients with no cancer [24].

Most interestingly, results of the studies of esophagus adenocarcinoma also showed that hTERT not only expressed in all cancer tissues but also in all adjacent non-cancerous tissues. Moreover, the trend toward longer telomeres with increasing depth of tumor invasion not only suggested for telomere lengths in cancer tissue but also for telomere Lengths in adjacent non-cancerous Barrett mucosa [25].

It is the first time report the positive rate of hTERT in peripheral blood mononuclear cells of the normal controls in our study. The mechanism is not clear.

The main discovery in the present study was EYA4 mRNA expression in peripheral blood mononuclear cells increased with the stages of progressive carcinogenesis of esophagus. Although the positive expression rates were relative low, using a positive cut-off value of 0.47, testing sensitivities were 4% and 16% for ESCD and ESCC, respectively, but the testing specificity increased to 100%, where no false positive cases were existed in the study.

Because there was a low degree of correlation between hTERT and EYA4 mRNA expression in the present study, both of them were dependent biomarkers. The discriminating ability between positive and negative status with either hTERT or EYA4 is too low to predict the high-risk persons.

In the study, we try to use the discriminating regression model to increase the power of predicting high-risk persons. Comparing with that in the discriminate models including independent variables of sex, age, smoking, drinking, family history of ESCC, in the model including the variables of hTERT, EYA4 and the five variables in the models increased the sensitivities and specificities of predicting ESCD and ESCC increased.

This knowledge may be useful in identifying high-risk persons who need to take part in the endoscopic test. It could be an effective method for monitoring the progress of premalignant esophageal lesions among subjects, who live in areas with high-incidence of cancer. A prospective study in the future needs to confirm these possibilities.

## Conclusion

In this study, we found out that the intensity of EYA4 and hTERT mRNA expression increases with the severity of esophageal pathological changes, which can bring forth values for monitoring the progress of premalignant esophageal lesions.

## Abbreviations

BCH: Basal cell hyperplasia; ESCD: esophageal squamous cells dyspalsia; ESCC: esophageal squamous cells cancer; EYA4: Eyes absent 4; hTERT: human telomerase reverse transcriptase.

## Competing interests

The authors declare that they have no competing interests.

## Authors' contributions

In our study all authors are in agreement with the content of the manuscript. Members listed below made their respective contributions to this manuscript. QHL, as correspondent author, study design and coordination, manuscript preparation. HL and TYD study design, experimental studies, data analysis, manuscript editing. ZYZ, FYY and QM study design and experiment of RT-PCR. All authors read and approved the final manuscript.
